# Editorial: Cerebellar structure and function in psychotic disorders: from mechanisms to clinics

**DOI:** 10.3389/fpsyt.2023.1344882

**Published:** 2023-12-07

**Authors:** Hengyi Cao, Ann K. Shinn, Wenbin Guo

**Affiliations:** ^1^Institute of Behavioral Science, Feinstein Institutes for Medical Research, Manhasset, NY, United States; ^2^Division of Psychiatry Research, Zucker Hillside Hospital, Glen Oaks, NY, United States; ^3^Department of Psychiatry, Zucker School of Medicine at Hofstra/Northwell, Hempstead, NY, United States; ^4^Schizophrenia and Bipolar Disorder Program, McLean Hospital, Belmont, MA, United States; ^5^Department of Psychiatry, Harvard Medical School, Boston, MA, United States; ^6^Department of Psychiatry, National Clinical Research Center for Mental Disorders and National Center for Mental Disorders, The Second Xiangya Hospital of Central South University, Changsha, China

**Keywords:** cerebellum, psychotic disorders, neuroimaging, neuromodulation, cognition

Research on cerebellar mechanisms in psychotic disorders has gained momentum in the past few years. A growing body of evidence has emerged to show the critical role of the cerebellum in cognition (1, 2) and pathogenesis of psychosis (3–6), and to demonstrate its potential value in treatment of schizophrenia (7, 8). In this Research Topic titled “*Cerebellar Structure and Function in Psychotic Disorders: From Mechanisms to Clinics*”, we collated a range of studies highlighting recent advances in the understanding of cerebellar structure and function in psychotic disorders. These studies, conducted primarily in patient samples, approached this topic by leveraging two major strategies commonly used in clinical neuroscience research, namely, neuroimaging and neuromodulation. Neuroimaging research utilizes techniques such as structural (sMRI), diffusional (dMRI), and functional magnetic resonance imaging (fMRI) to investigate gray matter morphometry, white matter microstructure, and neural activity and connectivity *in vivo*, linking their alterations to cognition and psychopathology. A more direct causal relationship from neural alterations to human behaviors can be informed by neuromodulation, including transcranial magnetic stimulation (TMS) as a widely used technique that regulates brain function by stimulation of a target region.

Three studies employed neuroimaging techniques to dissect region- and circuit-specific cerebellar abnormalities related to schizophrenia. Li et al. conducted a voxel-based meta-analysis to pinpoint the topography of cerebellar gray matter volume (GMV) changes in schizophrenia. Including a total of 25 studies comprising approximately 1,000 patients and 1,100 healthy subjects, they found that patients are associated with GMV reductions primarily in the left Crus II, right lobule VI, and right lobule VIII, three areas of the posterior cerebellum that have been linked to cognition in humans. Moreover, left Crus II GMV was negatively associated with patient age and illness duration, suggesting that this change may develop in tandem with illness progression, or reflect early neurodegeneration in the cerebellum in patients. These findings align well with the “cognitive dysmetria” hypothesis (9) that cognitive dysfunction may be an intermediate mechanism linking cerebellar abnormality and psychotic disorders. As another support for this hypothesis, Frosch et al. found that impaired social functioning (as measured by the Global Functioning Scale) in individuals at clinical high risk for psychosis was significantly correlated with reduced resting-state functional connectivity in Crus II and lobule VIII, the same cerebellar subfields reported in the above meta-analysis. This finding showed high regional specificity (i.e., was not observed in another control region, cerebellar lobule X) and was reversed in healthy subjects, suggesting that functional deficits in these specific cerebellar subfields may relate to difficulty in generating meaningful social behaviors and to future development of psychotic disorders. Together, these studies point to Crus II and lobule VIII as potential target areas for the treatment of cognitive and social deficits in schizophrenia.

The maintenance of the cerebellum's cognitive functions entails a delicate communication between the cerebellum and higher-order associative cortex in the cerebrum. Such communication is supported by white matter tracts, and deficits in cognitive functions may reflect alterations in white matter microstructure. To test this hypothesis, Chang et al. conducted a dMRI study to investigate voxel-wise changes in the cerebellar white matter and their associations with cognition in patients. The authors identified decreased fractional anisotropy (FA) and increased radial diffusivity (RD) mainly located at the left middle and inferior cerebellar peduncles, suggesting a potentially disrupted myelination in these peduncles. Notably, the FA and RD measures in these peduncles were found to be significantly associated with multiple cognitive domains including processing speed, working memory, and attention vigilance in healthy subjects but not patients, supporting the notion that intact microstructure of cerebellar white matter fibers is crucial to cognitive control in humans. However, due to the lack of direct association in the patient sample, it remains to be determined whether the detected alterations act as a causal mechanism for cognitive deficits or a secondary phenomenon emerged from the disorder or treatment. This calls attention to the need for more in-depth investigations of the cerebellum's input and output circuits, as proposed by Anteraper et al. in their perspective article. In the paper, the authors discussed the nuanced connections of the cerebellar-thalamo-cortical circuitry, with a particular focus on the dentate nuclei (DN), the primary output of the entire cerebellum. They argue that the DN can be functionally divided into three subfields, namely, the default-mode, salience-motor, and visual units. The detailed mapping of these functional units may add precision to the understanding of cerebellar connections and their function in psychiatric disorders, although more advanced imaging techniques such as 7-T MRI may be required to reach this goal.

On top of neuroimaging, two studies focusing on cerebellar TMS have brought this Research Topic closer to the clinic. Shinn et al. compared the effects of three commonly used cerebellar TMS protocols—intermittent (iTBS), continuous (cTBS), and sham theta burst stimulation—on timing-related cognition in patients with psychosis. By using a crossover design and an interval discrimination task, they found significantly reduced task reaction time after iTBS when compared with both cTBS and Sham, suggesting that the effect of cerebellar TMS on timing behaviors is dissociable and protocol-dependent. This study provides empirical evidence that may help guide the curation of cerebellar TMS protocol for future research and clinical use.

The choice of TMS protocol is only one of the many considerations in this research field. Hua et al. conducted a systematic review on present findings of cerebellar TMS in schizophrenia and discussed the current issues and obstacles to be overcome. With a total of 20 published studies, they found that cerebellar TMS is effective in the alleviation of negative and depressive symptoms, as well as increasing frontal-cerebellar connectivity in patients. Relatively less evidence was shown for cognitive improvement, which, however, may relate to the methodological issues they have discussed. These include, among others, the precise location of the stimulation, stimulus intensity, treatment length, how sham is defined, and issues of sample size and sample heterogeneity. It will not be until these questions are fully addressed that cerebellar TMS can be more effectively harnessed for clinical translation. Despite these limitations, this review presented data that clearly support the cerebellum as a promising neuromodulation target for the treatment of psychotic disorders.

In sum, the collection of articles in this Research Topic highlights the nuanced connections linking cerebellum, cognitive and related functions, and schizophrenia, while also highlighting the potential value of the cerebellum in the development of novel treatment strategies for schizophrenia.

## Author contributions

HC: Writing – original draft. AS: Writing – review & editing. WG: Writing – review & editing.
